# Interventions to Minimize Jet Lag After Westward and Eastward Flight

**DOI:** 10.3389/fphys.2019.00927

**Published:** 2019-07-31

**Authors:** Gregory D. Roach, Charli Sargent

**Affiliations:** Appleton Institute for Behavioural Science, Central Queensland University, Adelaide, SA, Australia

**Keywords:** time zone, adaptation, sleep, circadian, light, melatonin, exercise, athlete

## Introduction

Air travel across several time zones, i.e., transmeridian flight, causes negative effects—some of which occur during flight and some of which occur in the days after flight. Anecdotally, these effects are often referred to collectively as jet lag, but they are actually two separate phenomena—travel fatigue and jet lag—each with their own causes and consequences (Waterhouse et al., 2004). Travel fatigue refers to a collection of symptoms that occur during or immediately after long flights. These symptoms include fatigue, disorientation, and headache (Waterhouse et al., 2004)—primarily caused by the sleep loss, dehydration, hypoxia, and discomfort associated with being in an aircraft with confined space, recline-restricted seats, low air pressure, low humidity, etc., for 8–14 h (Brown et al., 2001; Roach et al., 2018). In contrast, jet lag refers to a collection of symptoms that occur in the days after flight across three or more time zones. These symptoms include headache, irritability, daytime sleepiness, difficulty sleeping at night, poor mental and physical performance, and poor gastrointestinal function (Waterhouse et al., 2004)—primarily caused by the mismatch between the circadian system, or internal body clock, which is synchronized to time cues in the departure time zone, and the desired timing of sleep and wake, which are typically synchronized to time cues in the destination time zone.

In August 2020, the Olympic Games will be held in Tokyo, Japan. Athletes will travel from all over the world to compete in the Games, and many will have to travel across several time zones. For example, athletes traveling to Japan from North America and Western Europe will face time zone changes of 8–11 h west and 6–8 h east, respectively. Some athletes will travel to Japan, or nearby countries, weeks before their events, while others will arrive in Japan in the days prior to competition. In either case, athletes will want to adjust to the new time zone as quickly as possible so that they can prepare well and/or compete at the highest level.

The purpose of this manuscript is to discuss the causes and consequences of jet lag and to provide examples of how to use judiciously timed light exposure/avoidance and/or exogenous melatonin ingestion to adapt the circadian system to a new time zone after transmeridian flight. These guides could be applied by athletes competing in the Tokyo 2020 Olympic Games, but they could also be applied by athletes traveling to other countries for training or competition, or by non-athletes traveling for business or pleasure.

## Jet Lag is Caused by the Desynchrony Between the Circadian System and Local Time Cues

The term “circadian rhythms” refers to rhythms with a period of approximately 24 h (Halberg, 1959). Many physiological and psychological variables in humans have been shown to alter rhythmically with a ~24 h period, including core body temperature (Dijk et al., 1992; Zhou et al., 2011a, 2017); cortisol (Scheer et al., 2009), blood pressure (Scheer et al., 2010), heart rate (Scheer et al., 2010), hunger (Sargent et al., 2016), cognitive performance (Dijk et al., 1992; Darwent et al., 2010; Matthews et al., 2010), strength (Reilly et al., 1997; Sargent et al., 2010), balance (Sargent et al., 2012b), flexibility (Reilly et al., 1997), dexterity (Matthews et al., 2012a,b), subjective alertness (Dijk et al., 1992; Zhou et al., 2012; Kosmadopoulos et al., 2014), subjective fatigue (Ferguson et al., 2012), subjective sleepiness (Kosmadopoulos et al., 2017), and objective sleepiness (Lavie, 1986; Dijk and Czeisler, 1995; Paech et al., 2010, 2012; Sargent et al., 2012a).

In humans, circadian rhythms in physiological and psychological variables are endogenously generated by a central circadian pacemaker—located within the suprachiasmatic nucleus of the hypothalamus—with a period of approximately 24.2 h (Czeisler et al., 1999; Zhou et al., 2011b). These rhythms are entrained to the period of a 24 h day by environmental signals or “zeitgebers,” meaning time-givers (Aschoff et al., 1971). Sunlight is the most powerful zeitgeber for humans (Wever et al., 1983; Czeisler et al., 1986), but non-photic stimuli such as social contact, eating, and physical activity, may also play a role (Mistlberger and Skene, 2004). Most peripheral cell types, including those in the major organ systems—heart, lungs, liver, pancreas—also contain their own circadian oscillators, which are kept in coherent phase relationships by the suprachiasmatic nucleus (Yamada and Forger, 2010; Bass, 2012; Schibler et al., 2015).

The circadian system cannot immediately entrain to the timing of zeitgebers in a new time zone (Wever, 1980), so after long-haul flights to the west or east, the circadian system is initially aligned with the timing of zeitgebers at the point of departure rather than zeitgebers at the new location (Winget et al., 1984). A period of desynchrony follows while the circadian system is entrained to the timing of zeitgebers in the new time zone—and it is this period of desynchrony that gives rise to the symptoms of jet lag.

## The Timing of the Human Circadian System Can be Reset by Light, Melatonin, and Exercise

**Figure 1:**
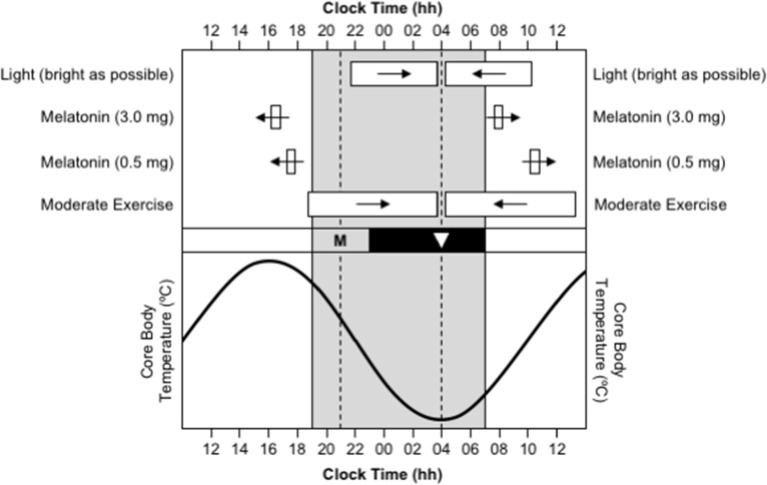
Use of appropriately timed stimuli to shift the timing of the human circadian system. In all sections, the gray background represents night-time. In the bottom section, the sinusoid-shaped line indicates the daily rhythm of core body temperature. In the middle section, the black bar represents habitual bedtime, “M” represents the evening onset of endogenous melatonin production, and the inverted triangle represents the daily minimum of core body temperature. In the top section, the white bars indicate the optimal timing of light exposure, exogenous melatonin ingestion, and moderate-intensity exercise, required to facilitate delays (right-pointing arrows) and advances (left-pointing arrows) in the circadian system. This figure was inspired by a similar figure presented by Waterhouse et al. (2007).

The timing of the human sleep/wake and circadian systems are related such that the production of endogenous melatonin begins ~2 h before habitual bedtime (Burgess et al., 2003b; Burgess and Eastman, 2005), the daily minimum of the core body temperature rhythm (CBTmin), which coincides with the daily low-point of the circadian cycle, occurs ~7 h after melatonin onset (Cagnacci et al., 1996; Brown et al., 1997; Eastman et al., 2000), and the daily peak of the core body temperature rhythm (CBTmax), which coincides with the daily high-point of the circadian cycle, occurs ~12 after CBTmin (Dijk et al., 1992). Therefore, a person who normally sleeps from 23:00 to 07:00 will have melatonin onset at ~21:00, CBTmin at ~04:00, and CBTmax at ~16:00 (Figure 1). Maximal sleepiness, and poorest mental/physical performance, occur in the 2–3 h either side of CBTmin, and maximal alertness, and greatest mental/physical performance, occur in the 2–3 h either side of CBTmax (Dijk et al., 1992). The protocols required to directly assess the timing of melatonin onset and CBTmin are invasive, time-consuming, and costly, so these variables are typically estimated based on the habitual timing of sleep and wake. However, work is currently being conducted to develop biomarkers of circadian phase based on the analysis of white blood cells from a single sample (Ueda et al., 2004; Wittenbrink et al., 2018).

Immediately after westward flight, the circadian system will be running ahead of the local time zone. For example, after a flight from London to Los Angeles (8 h west), when the body clock is ready for bed at 23:00 London time, it will only be 15:00 in Los Angeles. To adjust to the new time zone, the circadian system has to delay, or shift backward, or move later. Conversely, immediately after eastward flight, the circadian system will be running behind the local time zone. For example, after a flight from Los Angeles to London (8 h east), when it is time to get up at 07:00 in London, it is only 23:00 in Los Angeles, so the body clock will be ready for bed. To adjust to the new time zone, the circadian system has to advance, or shift forward, or move earlier.

Delays and advances in the timing of the circadian system can be facilitated by appropriately timed light exposure, melatonin ingestion, and/or exercise (Figure 1). The direction and size of the shift in the timing of the circadian system in response to these stimuli depend on the time of day, or more correctly, the circadian phase, that the stimuli occur. These effects are described by phase response curves (PRCs):

- Phase response curve to light. Light exposure in the ~12 h prior to CBTmin shifts the circadian system backward/later, or causes a phase delay; light exposure in the ~12 h after CBTmin shifts the circadian system forward/earlier, or causes a phase advance; and the largest shifts occur when light exposure occurs in the 3–6 h either side of CBTmin (Czeisler et al., 1989; Khalsa et al., 2003). Furthermore, the degree to which the timing of the circadian system can be shifted by exposure to light depends the duration of the exposure (Rimmer et al., 2000), the intensity of the light (Boivin et al., 1996), and the wavelength of the light (Wright and Lack, 2001; Rüger et al., 2013). The largest phase shifts occur when the duration of exposure is longer, when the intensity of light is higher, and when the wavelength of light is shorter (i.e., blue).
- Phase response curves to melatonin. Melatonin ingested in the late afternoon or early evening, a few hours prior to the onset of endogenous melatonin production and several hours prior to CBTmin, shifts the circadian system forward/earlier, or causes a phase advance (Burgess et al., 2008, 2010). Conversely, melatonin ingested in the morning, a few hours after habitual get-up time and several hours after CBTmin, shifts the circadian system backward/later, or causes a phase delay (Burgess et al., 2008, 2010). Separate PRCs have been created for exogenous melatonin at a “physiological” dose of 0.5 mg and at a “pharmacological” dose of 3.0 mg. For a 0.5 mg dose, it is estimated that maximum advances occur for ingestion ~10.5 h before CBTmin, or ~5.5 h before habitual bedtime, and maximum delays occur for ingestion ~6.5 h after CBTmin, or ~3.5 h after habitual get-up time (Burgess et al., 2010). For a 3.0 mg dose, it is estimated that maximum advances occur for ingestion ~11.5 h before CBTmin, or ~6.5 h before habitual bedtime, and maximum delays occur for ingestion ~4 h after CBTmin, or ~1 h after habitual get-up time (Burgess et al., 2008). The two doses of exogenous melatonin produce phase shifts of a similar size, but the 3.0 mg dose produces more reliable phase shifts than the 0.5 mg dose (Burgess et al., 2010). Goodness of fit data were not provided for either PRC, so the apparent precision of the estimated timing of melatonin ingestion for maximum advances and delays should be read with caution.
- Phase response curve to exercise. There is some evidence that exercise has phase-shifting properties (Eastman et al., 1995; Buxton et al., 2003), and a phase response curve to a 1 h bout of moderate-intensity exercise has been published recently (Youngstedt et al., 2019). The PRC indicates that exercise in the ~9 h prior to CBTmin shifts the circadian system backward/later, or causes a phase delay, and exercise in the ~9 h after CBTmin shifts the circadian system forward/earlier, or causes a phase advance. However, the protocol to obtain the data to construct this PRC was conducted with moderate-intensity light at 50 lux, instead of low-intensity light at <10–15 lux, so a PRC for the effects of exercise independent of light is still to be established.

This manuscript provides examples of how to use light and/or melatonin to shift the timing of the circadian system so that jet lag can be overcome as quickly as possible (**Figures 4**, **5**). Light and melatonin can be used independently, but their phase-shifting effects are additive—particularly for phase advances—so using them together should produce a greater effect than either one on its own (Wirz-Justice et al., 2004; Revell et al., 2006; Burke et al., 2013). In contrast, there is no evidence that the effects of light and exercise are additive, so exercise is not included in the adaptation guides. However, the appropriate times to conduct exercise for its phase-shifting properties coincide with the appropriate times for light exposure.

## The Experience of Jet Lag Depends on the Direction of Travel

**Figure 2:**
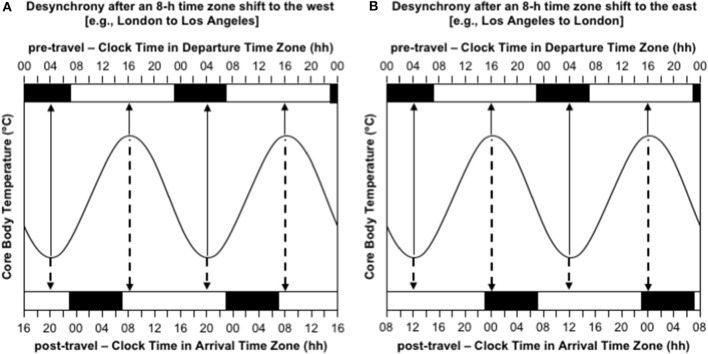
Desynchrony between the circadian system and the desired timing of sleep/wake immediately after time zone shifts to the west **(A)** and east **(B)**. In both panels, the sinusoid-shaped line represents core body temperature; the solid arrows indicate the timing of the daily minimum and maximum of core body temperature in the departure time zone; the dashed arrows indicate the timing of the daily minimum and maximum of core body temperature in the arrival time zone; the black bars at the top axis represent night-time sleep in the departure time zone; the black bars at the bottom axis represent night-time sleep in the arrival time zone; time is double-potted with 2 × 24 h periods. **(A)** Desynchrony after 8-h time zone shift to the west (e.g., London to Los Angeles). **(B)** Desynchrony after an 8-h time zone shift to the east (e.g., Los Angeles to London).

The most obvious consequences of jet lag are poor night-time sleep, excessive daytime sleepiness, and poor mental and physical performance (Waterhouse et al., 2004). However, the experience of jet lag greatly depends on the direction of travel. Consider the difference in the manifestation of jet lag between westward and eastward flights over 8 time zones—as occurs with travel between Western Europe (UTC+0 h) and the USA's west coast (UTC-8 h). Immediately after flying 8 h west, say from London to Los Angeles, the circadian system is still entrained to the timing of zeitgebers in London, so the daily low-point of the circadian cycle occurs at 04:00 London time, which is 20:00 in Los Angeles, and the daily high-point of the circadian cycle occurs at 16:00 London time, which is 08:00 in Los Angeles (Figure 2A). Consequently, at least initially in Los Angeles, a person will feel sleepy in the evening and they will have difficulty staying asleep until their normal wake time in the morning. If they are an athlete, they will also have difficulty training or competing at their highest level in the evening. In contrast, immediately after flying 8 h east, say from Los Angeles to London, the circadian system is still entrained to the timing of zeitgebers in Los Angeles, so the daily low-point of the circadian cycle occurs at 04:00 Los Angeles time, which is midday in London, and the daily high-point of the circadian cycle occurs at 16:00 Los Angeles time, which is midnight in London (Figure 2B). Consequently, at least initially in London, a person will feel sleepy in the late morning and early afternoon and they will have difficulty falling asleep at their normal bedtime in the evening. If they are an athlete, they will also have difficulty training and/or competing at their highest level in the late morning and early afternoon.

## Sunlight Can either Help or Hinder Adaptation to a New Time Zone

From anecdotal reports, it seems that a common perception among laypersons, is that maximizing exposure to sunlight in a new time zone is an effective strategy for overcoming jet lag. In general, this approach will work quite well after westward travel, but it may actually be counterproductive after eastward travel. To overcome jet lag, the circadian system must adjust so that it becomes aligned with the desired timing of sleep and wake in the new time zone. The most effective way to adjust the circadian system, or shift the timing of the body clock, is with exposure to light. However, light exposure, *per se*, does not cause the circadian system to align with the new time zone. Rather, light exposure shifts the timing of the body clock either backward (delay) or forward (advance), as required after westward and eastward travel, respectively.

**Figure 3:**
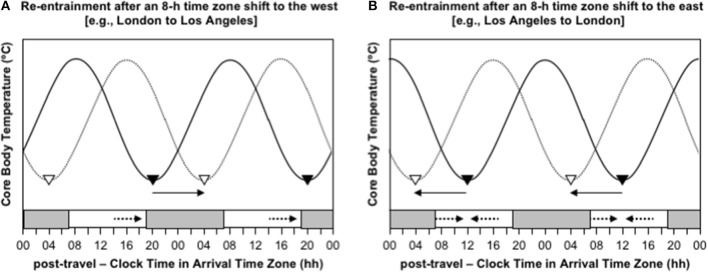
Re-entrainment of the circadian system to local time cues after time zone shifts to the west **(A)** and east **(B)**. In both panels, the solid sinusoid-shaped line with black inverted triangles represent core body temperature and the timing of its daily minimum immediately after flight; the dashed sinusoid-shaped line with white inverted triangles represent represents core body temperature and the timing of its daily minimum after re-entrainment to the arrival time zone; the gray bars at the bottom axis represent night-time in the arrival time zone; the solid arrows represent the direction of the phase shift required for adaptation to the new time zone; the dashed arrows represent the direction of the phase shift signal provided by light exposure at that time of day; time is double-potted with 2 × 24 h periods. **(A)** Re-entrainment after an 8-h time zone shift to the west (e.g., London to Los Angeles). **(B)** Re-entrainment after an 8-h time zone shift to the east (e.g., Los Angeles to London).

Consider the difference in the effectiveness of indiscriminately maximizing exposure to sunlight after westward and eastward travel over 8 time zones. Immediately after flying 8 h west, say from London to Los Angeles, the circadian system is still entrained to the timing of zeitgebers in London, so CBTmin occurs at 04:00 London time, which is 20:00 in Los Angeles. To adapt to the new time zone in Los Angeles, the circadian system must delay by 8 h, so that CBTmin occurs at the normal time of 04:00 instead of 20:00 (Figure 3A). If a person indiscriminately seeks sunlight during the daytime, they will be exposed to sunlight before CBTmin at 20:00, which will provide a delay signal, and they will be exposed to little or no sunlight after CBTmin, because the sun sets earlier than 20:00 in Los Angeles at most times of year, so they will not receive an advance signal (Figure 3A). In this case, maximizing exposure to sunlight would aid the desired phase delay. In contrast, immediately after flying 8 h east, say from Los Angeles to London, the circadian system is still entrained to the timing of zeitgebers in Los Angeles, so CBTmin occurs at 04:00 Los Angeles time, which is midday in London. To adapt to the new time zone in London, the circadian system must advance by 8 h, so that CBTmin occurs at the normal time of 04:00 instead of midday (Figure 3B). If a person indiscriminately seeks out sunlight during the daytime, they will be exposed to sunlight before CBTmin at midday, which will provide a delay signal, and they will be exposed to sunlight after CBTmin at midday, which will provide an advance signal (Figure 3B). In this case, maximizing exposure to sunlight would provide contradictory signals, which would inhibit adaptation to the new time zone. A better approach would be to avoid sunlight before midday and maximize exposure to sunlight in the 3–6 h after midday—that would limit the delay signal and maximize the desired advance signal.

## Endogenous Melatonin has Hypnotic and Chronobiotic Properties

Exogenous melatonin is both a chronobiotic and an hypnotoic, i.e., it can shift the timing of the circadian system (Arendt and Skene, 2005), but it can also make it easier to fall asleep and/or stay asleep (van den Heuvel et al., 2005; Zhdanova, 2005). The hypnotic effects of melatonin depend on the time of day, or more correctly, the circadian phase, that the melatonin is ingested (Wyatt et al., 2006). For exogenous melatonin, doses of ≤0.5 mg and 1–5 mg are typically considered to be physiological and pharmacological, respectively. If ingested when endogenous melatonin is high, i.e., during body clock night-time, neither a physiological 0.3 mg dose, nor a pharmacological 5.0 mg dose, of exogenous melatonin increase sleepiness. In contrast, if ingested when endogenous melatonin is low, i.e., during body clock daytime, both doses of exogenous melatonin increase sleepiness to a similar extent. Therefore, if melatonin is used as a chronobiotic at either physiological or pharmacological doses, its potential hypnotic effects should also be considered. This manuscript provides advice on how to exploit the chronobiotic effects of a pharmacological 3.0 mg dose, rather than a physiological 0.5 mg dose, because the higher dose produces more reliable phase shifts than the lower dose (Burgess et al., 2010).

## Schedules for Shifting the Timing of the Circadian System After Transmeridian Air Travel

After long-haul flights across multiple time zones, the circadian system is initially aligned with the timing of zeitgebers at the point of departure rather than zeitgebers at the new location. To overcome jet lag, the timing of the circadian system must shift so that it becomes aligned with the new time zone. Adaptation guides have previously been presented for relatively large time zone shifts of 7–9 h (Eastman and Burgess, 2009; Revell and Eastman, 2012). The following sub-sections provide examples of how to use light and/or melatonin to shift the timing of the circadian system so that jet lag can be overcome as quickly as possible after rapid time zone changes of 3, 6, 9, and 12 h to the west and east.

**Figure 4:**
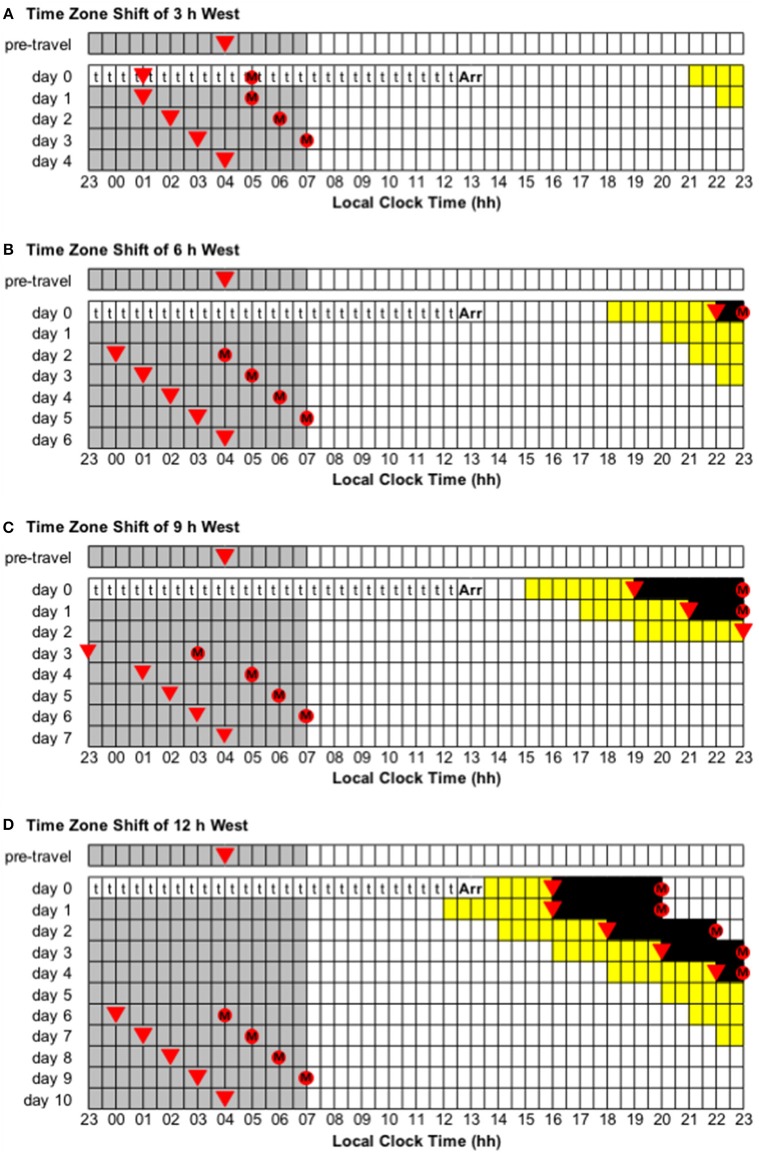
Use of light exposure/avoidance and ingestion of exogenous melatonin to facilitate adaptation to westward time zone shifts of 3, 6, 9, and 12 h. In all panels, “t” represents transmeridian travel; “Arr” represents arrival in the new time zone; gray boxes represent sleep; white boxes represent wake; yellow boxes represent light exposure during wake; black boxes represent light avoidance during wake; red circles with an “M” represent ingestion of a 3.0 mg dose of exogenous melatonin; inverted triangles represent the daily minimum of core body temperature (CBTmin). Partial adaptation is achieved when CBTmin in the new time zone occurs within the scheduled sleep period, and complete adaptation is achieved when CBTmin in the new time zone occurs at the same time as the pre-travel CBTmin. On days when the ideal time of melatonin ingestion occurs during a scheduled sleep period, it should occur at that time if awake, otherwise it should be missed. This figure was inspired by similar figures presented by Eastman and Burgess (2009) and Revell and Eastman (2012). **(A)** Time zone shift of 3 h west. **(B)** Time zone shift of 6 h west. **(C)** Time zone shift of 9 h west. **(D)** Time zone shift of 12 h west.

**Figure 5:**
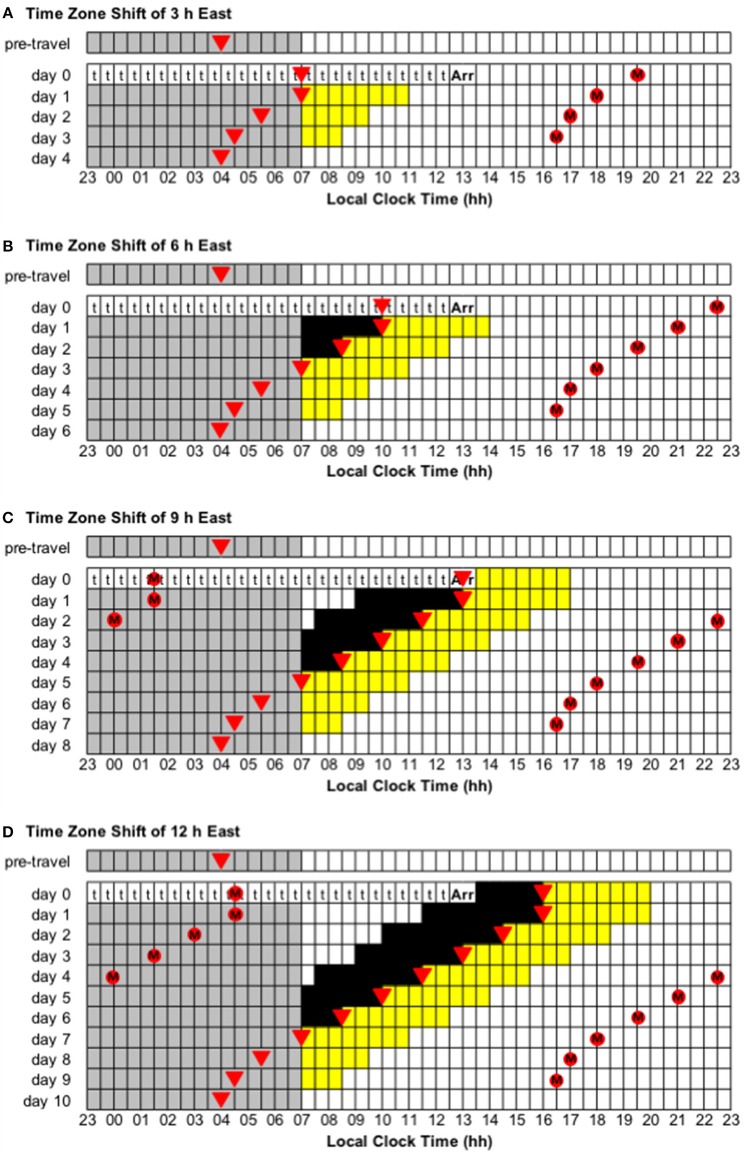
Use of light exposure/avoidance and ingestion of exogenous melatonin to facilitate adaptation to eastward time zone shifts of 3, 6, 9, and 12 h. In all panels, “t” represents transmeridian travel; “Arr” represents arrival in the new time zone; gray boxes represent sleep; white boxes represent wake; yellow boxes represent light exposure during wake; black boxes represent light avoidance during wake; red circles with an “M” represent ingestion of a 3.0 mg dose of exogenous melatonin; inverted triangles represent the daily minimum of core body temperature (CBTmin). Partial adaptation is achieved when CBTmin in the new time zone occurs within the scheduled sleep period, and complete adaptation is achieved when CBTmin in the new time zone occurs at the same time as the pre-travel CBTmin. On days when the ideal time of melatonin ingestion occurs during a scheduled sleep period, it should occur at that time if awake, otherwise it should be missed. This figure was inspired by similar figures presented by Eastman and Burgess (2009) and Revell and Eastman (2012). **(A)** Time zone shift of 3 h east. **(B)** Time zone shift of 6 h east. **(C)** Time zone shift of 9 h east. **(D)** Time zone shift of 12 h east.

The adaptation guides are based on three major assumptions:

- Assumption 1—A person who normally sleeps from 23:00 to 07:00 will have the daily minimum of their core body temperature rhythm (CBTmin) at ~04:00. For people with earlier or later bedtimes, the timing of light and melatonin should be adjusted accordingly. For example, a person who normally sleeps from 22:00 to 06:00 will have CBTmin at ~03:00 instead of ~04:00, so the timing of light and melatonin should be 1 h earlier than in the examples, and a person who normally sleeps from midnight to 08:00 will have CBTmin at ~05:00 instead of ~04:00, so the timing of light and melatonin should be 1 h later than in the examples.
- Assumption 2—A person wants their main daily sleep period to occur at the same local time in the arrival time zone as in their normal time zone (i.e., 23:00 to 07:00 in the examples provided). In some cases, this may be difficult to achieve in practice, particularly on the first few days after travel. For example, after a shift of 9 time zones west (Figure 4C), a person may be sleepy in the evening, so they may wish to go to bed earlier than usual (e.g., 21:00–05:00 instead of 23:00–07:00). Similarly, after a shift of 9 time zones east (Figure 5C), a person may not be sleepy in the evening, so they may wish to go to bed later than usual (e.g., 01:00–09:00 instead of 23:00–07:00). In situations where the timing of the main sleep period differs from the timing in the relevant guide, this should not interfere with adaptation provided that light exposure/avoidance and/or melatonin ingestion still occur at the appropriate time.
- Assumption 3—Arrival in the new time zone occurs at 13:00 local time. In certain cases where arrival is earlier or later, it may be necessary for the adaptation schedule to be advanced or delayed by a day, respectively. For example, for a 21:00 arrival after a 9 h westward time zone change, the day 0 schedule should be delayed to occur on day 1 because arrival would occur after the critical time for light exposure, so adaptation has to begin one day “late” (Figure 4C). Conversely, for a 06:00 arrival after a 6 h eastward time zone change, the day 1 schedule should be advanced to occur on day 0 because arrival would occur prior to the critical time for light exposure, so adaptation can begin one day “early” (Figure 5B).

When a schedule requires light exposure during the daytime, it is best to be outside in sunlight without sunglasses. When a schedule requires light exposure after sunset, bright indoor light, or a light box, or light-emitting glasses should be used. When a schedule requires light avoidance, it is best to be inside with lights off or as dim as possible—it may even be appropriate to have a nap (limited to 1 h so as not to interfere with night-time sleep). When a schedule requires light avoidance during the daytime and being outside is unavoidable, wrap-around sunglasses with minimal light transmission should be worn.

Complete adaptation after transmeridian flight is achieved when the circadian system shifts sufficiently such that CBTmin in the arrival time zone occurs at the same time as it occurred in the departure time zone (i.e., 04:00, assuming habitual bed time of 23:00–07:00). However, this can take several days, so from a practical point of view, it is most important that the circadian system shifts sufficiently such that CBTmin occurs during the main night-time sleep period in the new time zone—a state of partial adaptation (Eastman and Burgess, 2009). Once CBTmin—the daily low-point of the circadian cycle—occurs during the night-time, sleep should be longer and of better quality, daytime sleepiness should be reduced, and mental and physical performance should be higher, i.e., the symptoms of jet lag should be greatly reduced. This distinction between complete and partial adaptation has previously been applied to shift workers switching from day work to night work (Lee et al., 2006).

NB. If melatonin is used by an athlete, a responsible party must ensure that it is not a prohibited substance under the relevant drug code and that it is sourced from a reputable supplier (to ensure purity and dose accuracy).

Adaptation schedules after westward flight:

- Time zone change of 3 h west (e.g., from Wellington, New Zealand, to Tokyo, Japan). Immediately after flight, CBTmin will occur at 01:00 local time, instead of 04:00, and CBTmax will occur at 13:00 local time, instead of 16:00. To adapt to the new time zone, the circadian system has to delay by 3 h—partial and complete adaptation should be achieved on days 1 and 4, respectively (Figure 4A).
- Time zone change of 6 h west (e.g., from Anchorage, USA, to Tokyo, Japan). Immediately after flight, CBTmin will occur at 22:00 local time, instead of 04:00, and CBTmax will occur at 10:00 local time, instead of 16:00. To adapt to the new time zone, the circadian system has to delay by 6 h—partial and complete adaptation should be achieved on days 2 and 6, respectively (Figure 4B).
- Time zone change of 9 h west (e.g., from Minneapolis, USA, to Tokyo, Japan). Immediately after flight, CBTmin will occur at 19:00 local time, instead of 04:00, and CBTmax will occur at 07:00 local time, instead of 16:00. To adapt to the new time zone, the circadian system has to delay by 9 h—partial and complete adaptation should be achieved on days 3 and 7, respectively (Figure 4C).

Adaptation schedules after eastward flight:

- Time zone change of 3 h east (e.g., from Dhaka, Bangladesh, to Tokyo, Japan). Immediately after flight, CBTmin will occur at 07:00 local time, instead of 04:00, and CBTmax will occur at 19:00 local time, instead of 16:00. To adapt to the new time zone, the circadian system has to advance by 3 h—partial and complete adaptation should be achieved on days 1 and 4, respectively (Figure 5A).
- Time zone change of 6 h east (e.g., from Doha, Qatar, to Tokyo, Japan). Immediately after flight, CBTmin will occur at 10:00 local time, instead of 04:00, and CBTmax will occur at 22:00 local time, instead of 16:00. To adapt to the new time zone, the circadian system has to advance by 6 h—partial and complete adaptation should be achieved on days 3 and 6, respectively (Figure 5B).
- Time zone change of 9 h east (e.g., from London, United Kingdom, to Tokyo, Japan). Immediately after flight, CBTmin will occur at 13:00 local time, instead of 04:00, and CBTmax will occur at 01:00 local time, instead of 16:00. To adapt to the new time zone, the circadian system has to advance by 9 h—partial and complete adaptation should be achieved on days 5 and 8, respectively (Figure 5C).

Adaptation schedules after a time zone change of 12 h east/west (e.g., from Buenos Aires, Argentina, to Tokyo, Japan):

- Immediately after flight, CBTmin will occur at 16:00 local time, instead of 04:00, and CBTmax will occur at 04:00 local time, instead of 16:00. To adapt to the new time zone, the circadian system could either delay or advance by 12 h. However, given that the human circadian system has a natural period of ~24.2 h such that it has a greater propensity to delay than to advance (Czeisler et al., 1999; Zhou et al., 2011b), it is more common to adapt by delay. Using a delay schedule, partial and complete adaptation should be achieved on days 6 and 10, respectively (Figure 4D). Using an advance schedule, partial and complete adaptation should be achieved on days 7 and 10, respectively (Figure 5D).

## Evidence of Efficacy for Jet Lag Interventions Based on Light and/or Melatonin

This manuscript contains recommendations regarding the use of judiciously timed light exposure/avoidance and ingestion of exogenous melatonin to minimize jet lag by facilitating adaptation of the circadian system to a new time zone. These recommendations are primarily based on information contained in phase response curves, which describe the effects of light and melatonin on the timing of the circadian system (Czeisler et al., 1989; Khalsa et al., 2003; Burgess et al., 2008, 2010). Computer-based simulations with experimentally-validated mathematical models have demonstrated that schedules of light exposure and avoidance could be used to increase the rate of adaptation after a rapid shift in the timing of the light-dark cycle (Serkh and Forger, 2014). Laboratory-based trials have established that both light and melatonin, when administered alone, can shift the timing of the circadian system (Deacon and Arendt, 1995; Middleton et al., 1997; Burgess et al., 2003a; Smith and Eastman, 2009), and when used in combination, the phase-shifting effects of light and melatonin are additive (Revell et al., 2006; Paul et al., 2011). Only a few field studies have been conducted to examine the efficacy of light-based interventions for the treatment of jet lag—and their results are equivocal (Boulos et al., 2002; Lahti et al., 2007; Thompson et al., 2012). In contrast, a meta-analysis of 10 field studies examining the efficacy of melatonin-based interventions indicates that they are effective at shifting the body clock and at reducing subjective ratings of jet lag (Herxheimer and Petrie, 2002). To date, the efficacy of combined light/melatonin interventions has not been assessed in field-based settings, so this is a critical next step for the advancement of knowledge in this field.

## Could Adaptation Begin Before/During Transmeridian Travel?

Specific guides have not been provided here, but it is possible to begin shifting the circadian system in the desired direction before and/or during transmeridian flight (see Eastman and Burgess, 2009; Revell and Eastman, 2012). The potential advantage of pre-shifting is that it should reduce the amount of time required to adapt to local time cues in the arrival time zone, such that the symptoms of jet lag are less pronounced and/or occur over fewer days. Conversely, the potential disadvantages of pre-shifting are that it could interfere with sleep and be socially disruptive prior to travel and it could make it more difficult to estimate the timing of the daily minimum in core body temperature (CBTmin), and thus the appropriate times for light exposure/avoidance and/or melatonin ingestion, upon arrival in the new time zone.

To delay the circadian system in the 3–4 days prior to westward travel, gradually move bedtime and get-up time later (i.e., 30–60 min per day), maximize evening light exposure, minimize morning light exposure, and take 3.0 mg of melatonin 1 h after rising from bed. To advance the circadian system in the 3–4 days prior to eastward travel, gradually move bedtime and get-up time earlier (i.e., 30–60 min per day), minimize evening light exposure, maximize morning light exposure, and take 3.0 mg of melatonin 6.5 h before bed.

To shift the circadian system during travel, rather than setting a watch and attempting to align sleep and wake with the arrival time zone, light exposure/avoidance and melatonin ingestion should be timed according to the departure time zone. To delay the circadian system during westward travel, maximize light exposure in the ~3 h before CBTmin, avoid light in the ~3 h after CBTmin (by sleeping if possible), and take 3.0 mg of melatonin 4 h after CBTmin. To advance the circadian system during eastward travel, take 3.0 mg of melatonin 11.5 h before CBTmin, avoid light in the ~3 h prior to CBTmin (by sleeping if possible), and maximize light exposure in the ~3 h after CBTmin.

## Conclusions

Long-haul flight over several time zones causes both travel fatigue and jet lag. The most obvious consequences of jet lag are poor sleep at night, excessive sleepiness during the day, and poor mental and physical performance. These consequences occur because the human circadian system cannot immediately adapt to time cues in a new time zone. This manuscript has presented recommendations on how to minimize jet lag using judiciously timed light exposure/avoidance and ingestion of exogenous melatonin to facilitate adaptation of the circadian system to a new time zone. These recommendations are based on the latest information regarding the effects of light and melatonin on the human circadian system. There are potential barriers to the practical implementation of these recommendations, so it will be critical to assess their efficacy in natural settings, preferably using experimental designs with randomization to treatment and control groups.

## Author Contributions

GR and CS contributed to conception of the manuscript. GR wrote the first draft of the manuscript. GR and CS contributed to revision of the manuscript. GR and CS read and approved the submitted version of the manuscript.

### Conflict of Interest Statement

The authors declare that the research was conducted in the absence of any commercial or financial relationships that could be construed as a potential conflict of interest.
